# Supplementary material for: Surface Vertical Multi-Emission Laser with Distributed Bragg Reflector Feedback from CsPbI3 Quantum Dots
Source: Nanomaterials (Basel). 2023 May 18;13(10):1669. doi: 10.3390/nano13101669 (PMC10222913; doi:10.3390/nano13101669)
Supplement: Supplementary file 1 [file nanomaterials-13-01669-s001.zip › nanomaterials-2323960-supplementary.pdf]

nanomaterials

Supplementary Information

Surface Vertical Multi-Emission Laser with Distributed Bragg Reflector Feedback from $CsPbI_3$ Quantum Dots

Xueqiong Su [1,*,†], Yong Pan [2,†], Dongwen Gao [1], Jin Wang [1], Huimin Yu [1], Ruixiang Chen [1], Baolu Guan [3], Xinyu Yang [4], Yimeng Wang [5] and Li Wang [1,*]

1 College of Physics and Optoelectronics, Faculty of Science, Beijing University of Technology, Beijing 100124, China
2 College of Science, Xi'an University of Architecture and Technology, Xi'an 710055, China
3 Key Laboratory of Opto-Electronics Technology, Ministry of Education, Faculty of Information Technology, Beijing University of Technology, Beijing 100124, China
4 The College of Chemistry & Materials Engineering, WenZhou University, Wenzhou 325000, China
5 The School of Optical-Electrical and Computer Engineering, University of Shanghai for Science and Technology, Shanghai 200093, China
* Correspondence: nysxq@bjut.edu.cn (X.S.); lwang.1@bjut.edu.cn (L.W.)
† These authors contributed equally to this work.

S1. The details of sputtering

Table S1. The details of sputtering.

Process parameters

Background vacuum	2.5×10^{-4} Pa
Heating temperature	100℃
Pre sputtering time	300s
Intermittent circulation	300s
Number of cycles	5(6)

TiO_2

RF power	200 W
Gas flow	Ar, 60 sccm
Process time	1792 s

SiO_2

RF power	300 W
Gas flow	Ar, 90 sccm
Process time	1133 s

S2. The video of QDs preparation

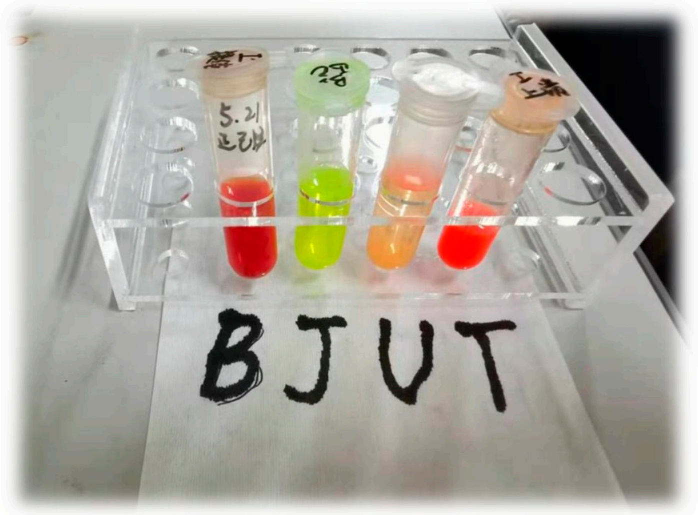

Figure S1 Physical demonstration of quantum dots and PL emission video. In this work, the composition changes from $CsPb(Br_{1-x}Cl_x)_3$ to $CsPb(Br_{1-y}I_y)_3$. The green quantum dot is a Br dominated system, and the red quantum dot is an I dominated system.

The video of the quantum dot production process was uploaded as a supplementary file. It is shown in the video that quantum dots can generate emission at corresponding wavelengths when excited by a UV 365 nm fluorescent light source.

S3. The physical photograph of DBR laser

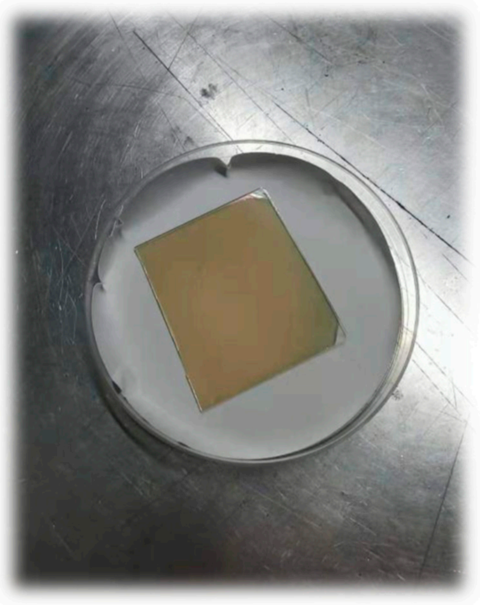

Figure S2 The physical photograph of lower layer of DBR laser.

The physical photograph of lower layer in DBR laser with 1×1 cm^2 substrate. The DBR lasers are prepared by magnetron sputtering (MSP-3200). Sputtering refers to the phenomenon that particles with a certain amount of energy bombard the surface of a solid, causing solid molecules or atoms to leave the solid and eject from

the surface. Sputtering coating refers to the process of using the sputtering effect generated by particles bombarding the target material to cause the target atoms or molecules to shoot out from the solid surface, and depositing a thin film on the substrate. Magnetron sputtering is the introduction of a magnetic field between the two electrodes of glow discharge. The electrons are accelerated by the electric field while being bound by the magnetic field. The motion trajectory becomes a cycloid, increasing the probability of electrons colliding with charged particles and gas molecules, increasing the ionization rate of the gas, and reducing the working pressure. Under the acceleration of a high-voltage electric field, Ar^{+} ions collide with the target material and release energy. The target atoms on the surface of the target material escape from the target material and fly towards the substrate, and are deposited on the substrate to form a thin film.

S4. The FDTD model for simulation

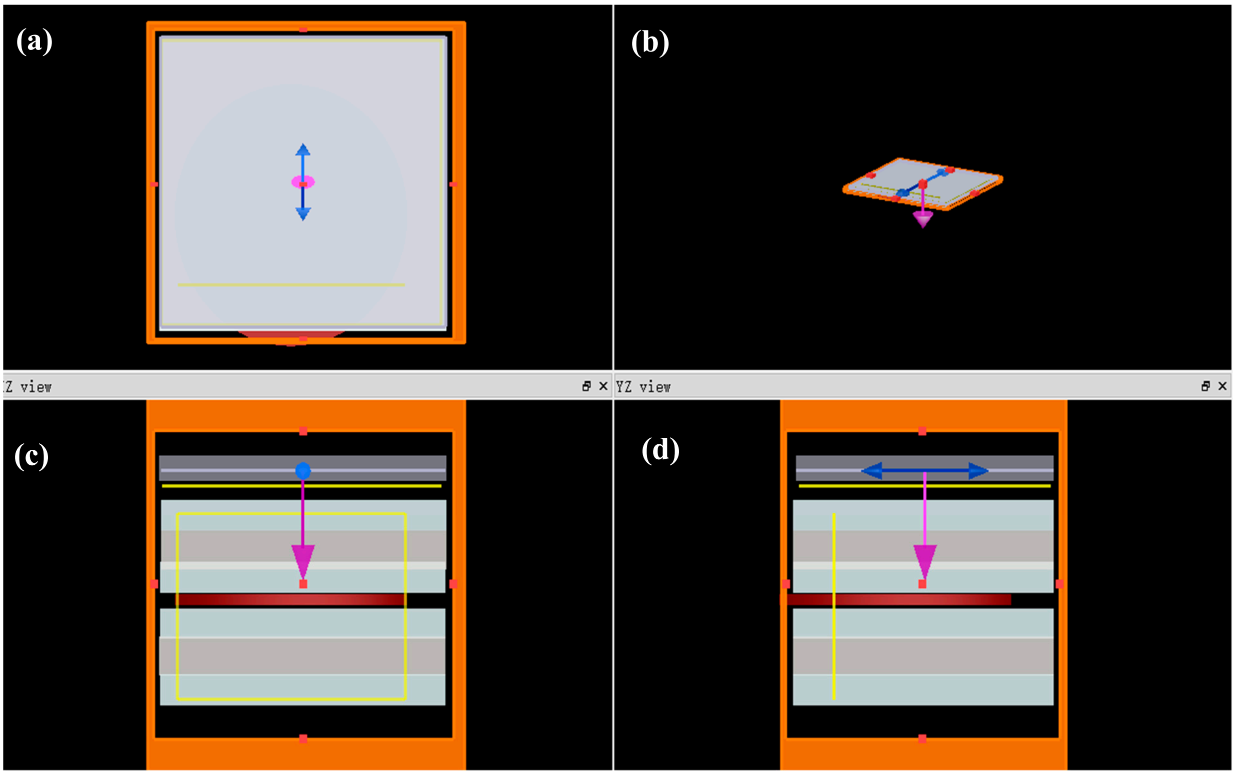

Figure S3 The FDTD model image for simulation. (a) XY view of the DBR model; (b) Axial view of the DBR model; (c) XZ view of the DBR model; (d) YZ view of the DBR model.

The DBR model established by Finite Difference Time Domain (FDTD), which is a simulation of 3D/2D Maxwell's Solver for Nanophotonic Devices. Here, the establishment of our DBR model is consistent with its physical dimensions. FDTD is the gold-standard for modeling nanophotonic devices, processes, and materials. This finely-tuned implementation of the FDTD method delivers reliable, powerful, and scalable solver performance over a broad spectrum of applications. The integrated design environment provides scripting capability, advanced post-processing, and optimization routines – allowing you to focus on your design and leave the rest to software.

The basic idea of the FDTD method is to replace the first partial derivative of the field in time and space with the central difference quotient, and then the field distribution can be obtained by recursively simulating the

wave propagation process in time domain. FDTD directly discretizes the wave equation in time domain. It does not need any form of derived equation, so it will not limit its application because of the mathematical model. The difference scheme contains the parameters of the medium, and it can simulate various complex structures only by giving the corresponding parameters to each grid. This is an outstanding advantage of the FDTD method. In addition, because the finite-difference time-domain method uses the step-by-step method, it is easy to simulate various complex broadband signals in time domain, and it is very convenient to obtain the time-domain signal waveform at a certain point in space.

S5. The FDTD model data for simulation

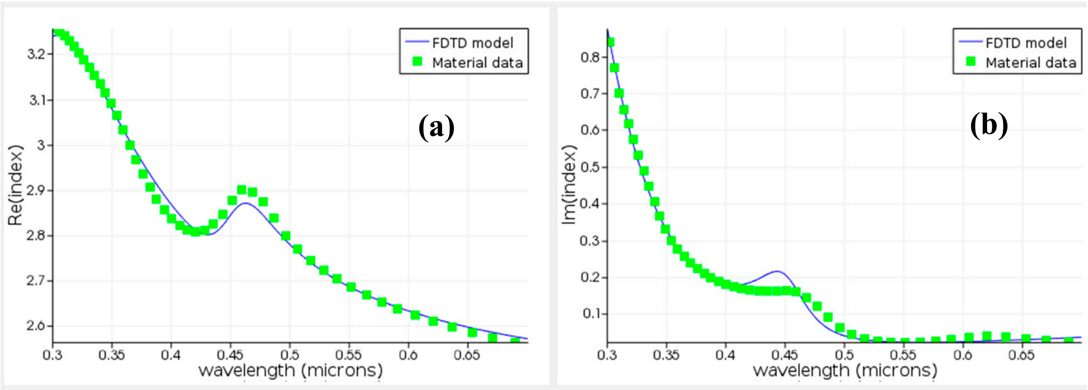

Figure S4 The FDTD model data for simulation. (a) The real part of the refractive index. (b) The imaginary part of refractive index.

The data of our materials is tested by step apparatus. We added the data of perovskite QDs to the FDTD materials library. From the fitting results, the theoretical and actual values of the material are in good agreement to $CsPbI_3$ QDs.
